# Supplementary material for: Identification and characterization of CIM-1, a carbapenemase that adds to the family of resistance factors against last resort antibiotics
Source: Commun Biol. 2024 Mar 7;7:282. doi: 10.1038/s42003-024-05940-0 (PMC10920655; doi:10.1038/s42003-024-05940-0)
Supplement: Supplementary file 2 — Supplementary Information [file 42003_2024_5940_MOESM2_ESM.pdf]

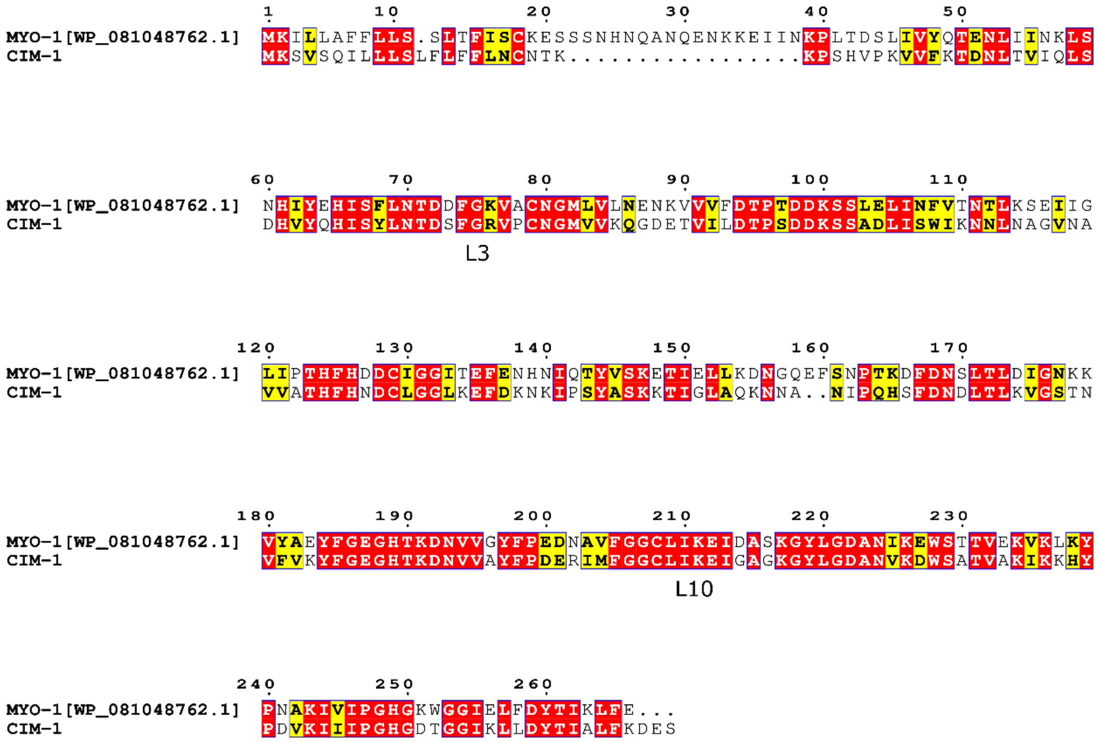

Supplementary Figure 1. The top protein selected by SWISS-MODEL during modelling of CIM-1 3D structure, MYO-1, has a high amino acid similarity comparing to CIM-1. Non-canonical lipobox (FISC) was also observed from MYO-1 amino acid sequence at position 15 to 18. MYO-1 was predicted by SignalP6.0 to be a lipoprotein at a likelihood of 0.8233. Sequence identity and similarity was determined using Sequence Manipulation Suit (SMS): identical residues (denoted by a red box): 132; similar residues (denoted by a yellow box): 46; percent identity:48.89; percent similarity: 65.93. Amino acid sequence alignment of CIM-1 with NDM-1 was generated by ESPript 3.0.

Sequence
Prediction: Lipoprotein signal peptide (Sec/SPII)

Cleavage site between pos. 18 and 19. Probability 0.980659

Protein type	Other	Signal Peptide (Sec/SPI)	Lipoprotein signal peptide (Sec/SPII)	TAT signal peptide (Tat/SPI)	TAT Lipoprotein signal peptide (Tat/SPII)	Pilin-like signal peptide (Sec/SPIII)
**Likelihood**	0	0.0091	0.991	0	0	0

Download: PNG / EPS / Tabular

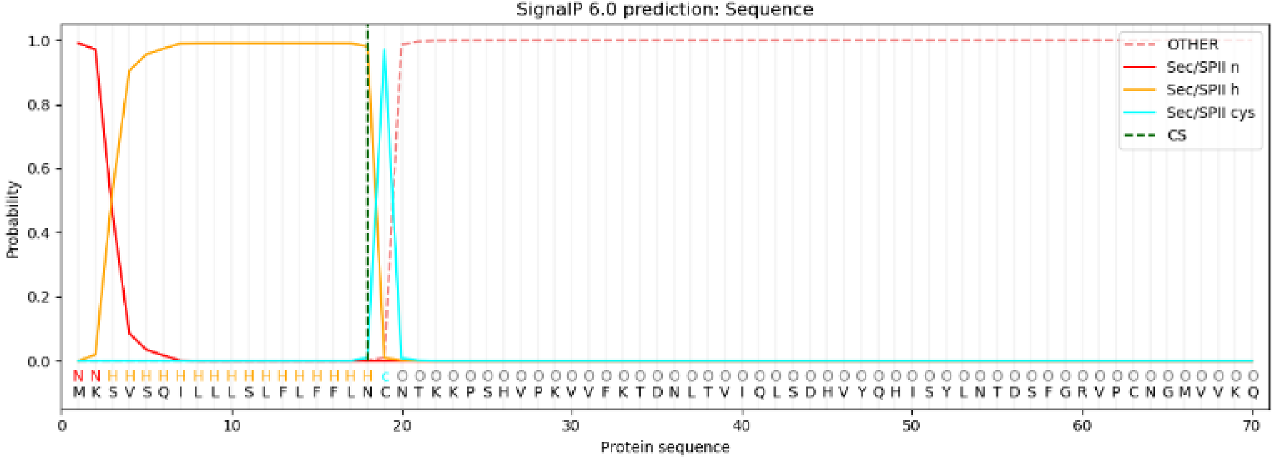

Supplementary Figure 2. CIM-1 was predicted as lipoprotein at a high likelihood (0.991). The signal peptide cleavage site was predicted between position 18 and 19.

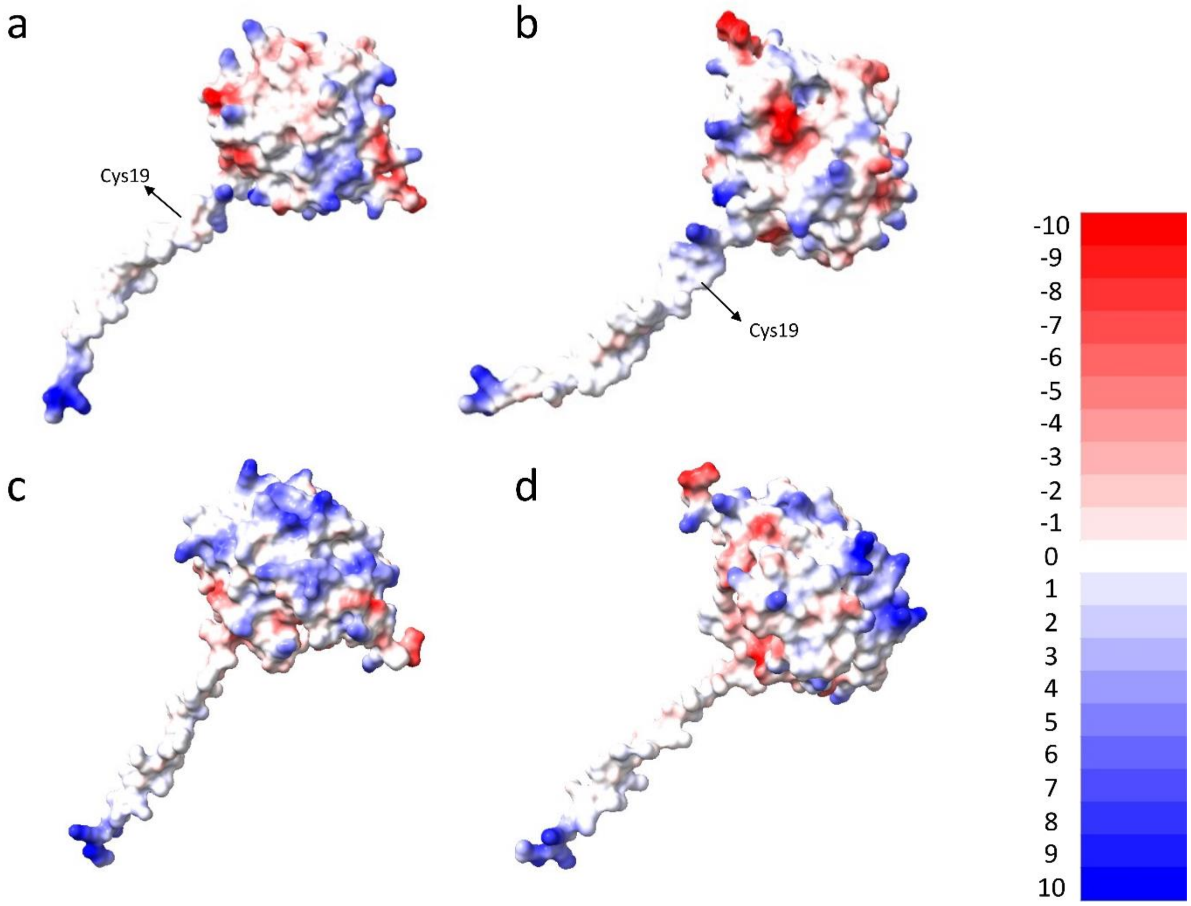

Supplementary Figure 3. a-b) Electrostatic surface potential of CIM-1. c-d) Electrostatic surface potential of IND-2. Protein structure was fetched using Alphafold in ChimeraX (v 1.6.1). Electrostatic surface potential was generated using ChimeraX surface colour function.

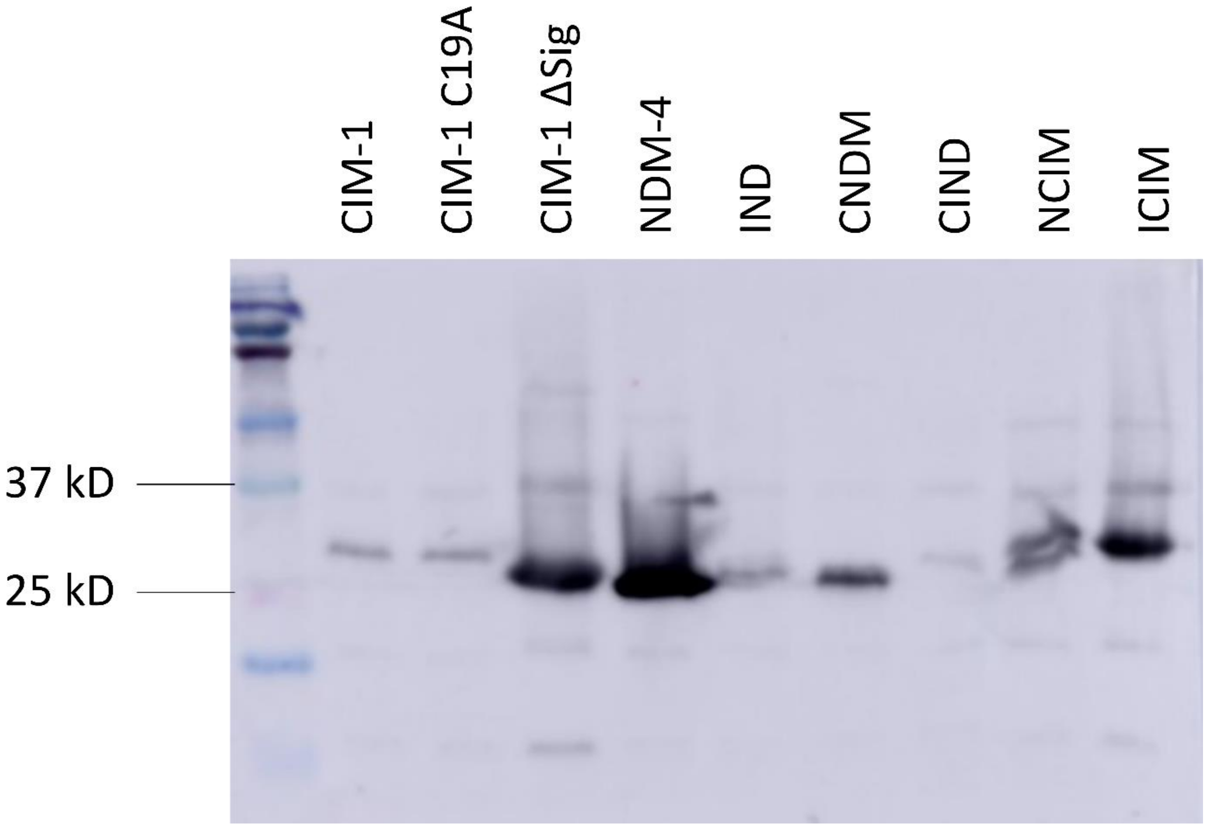

Supplementary Figure 4. Relative expression level of all His-tagged constructs (CIM-1, IND-2, NDM-4, CIM-1ΔSig, CIM-1 C19A, I-CIM, N-CIM, C-NDM and C-IND) in *E. coli* C41(DE3) cells. Cells were inoculated from an overnight culture and grown the same manner as preparation for antimicrobial susceptibility assay.

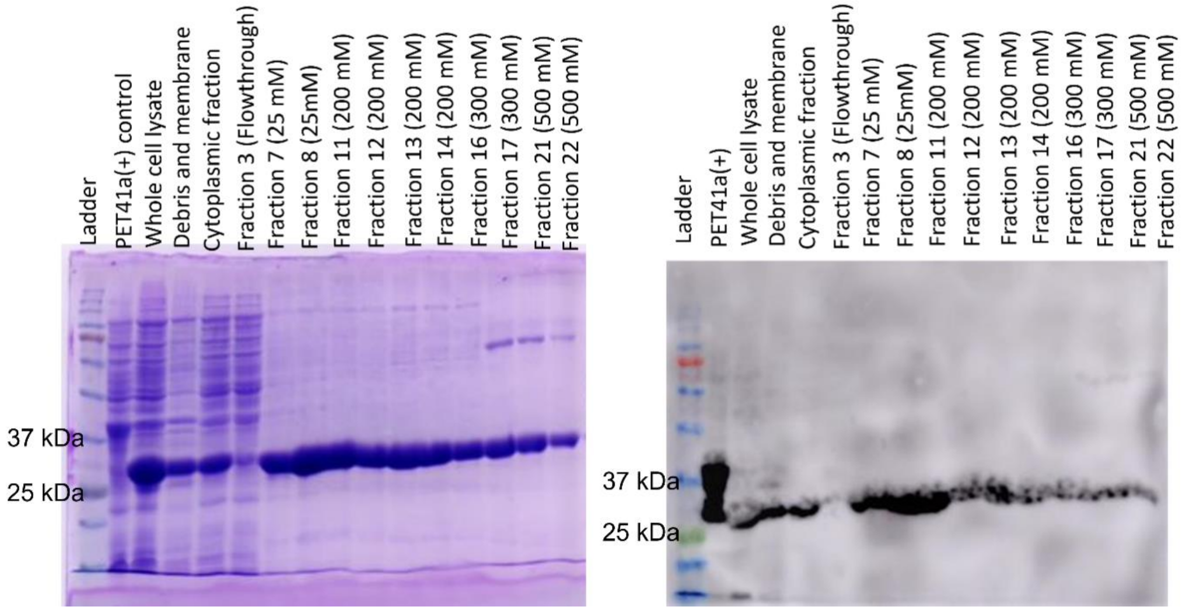

Supplementary Figure 5. SDS-PAGE analysis of His-tagged CIM-1ΔSig protein purification.

E. coli BL21(DE3) was transformed with pET41a(+)-*bla*$_{\text{CIM-1ΔSig}}$ and grown in LB at 25 °C for 20 hours. The protein was purified from the supernatant after ultra-centrifugation of disrupted cells using an ÄKTA pure FPLC and Nickel-affinity column chromatography. Peaks across all fractions were loaded onto 15% SDS-PAGE gel. Proteins were visualized via gel staining with Coomassie® Brilliant Blue R-250 (Bio-Rad). High purity of CIM-1ΔSig was observed across most fractions due to its high expression level. Western blot was used to confirm His-tagged CIM-1ΔSig at approximately 27 kDa (Theoretical weight of His-tagged CIM-1ΔSig is 26908.49 Da, calculated using Expasy pI tool: https://web.expasy.org/compute_pi/)

MKSVSQILLLSLFLFFLN/CNTKKPSHVPKVVFKTDNLTVIQLSDHVYQHISYLNTDSFGRV
PCNGMVVKQGDETVILDTPSDDKSSADLISWIKNNLNAGVNAVVATHFHNDCLGGLKE
FDKNKIPSYASKKTIGLAQKNNANIPQHSFDNDLTLKVGSTNVFVKYFGEGHTKDNVVAY
FPDERIMFGGCLIKEIGAGKGYLGDANVKDWSATVAKIKKHYPDVKIIIPGHGDTGGIKLL
DYTIALFKDESHHHHHH

Supplementary Figure 6. His-tagged CIM-1 sequence. Peptide sequences identified from LC-MS/MC analysis were in grey shades. Predicted signal peptide cleavage site was indicated as "/".

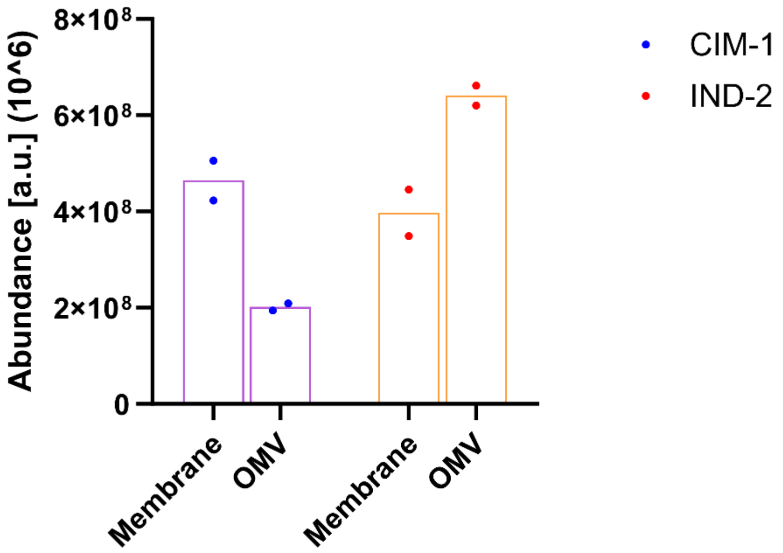

Supplementary Figure 7. Protein abundance of CIM-1 and IND-2 in membrane fraction and outer membrane vesicles samples of *C. indologenes.* Individual data points shown are two technical duplicates from Mass spectrometry analysis. Error bars were shown for SEM.

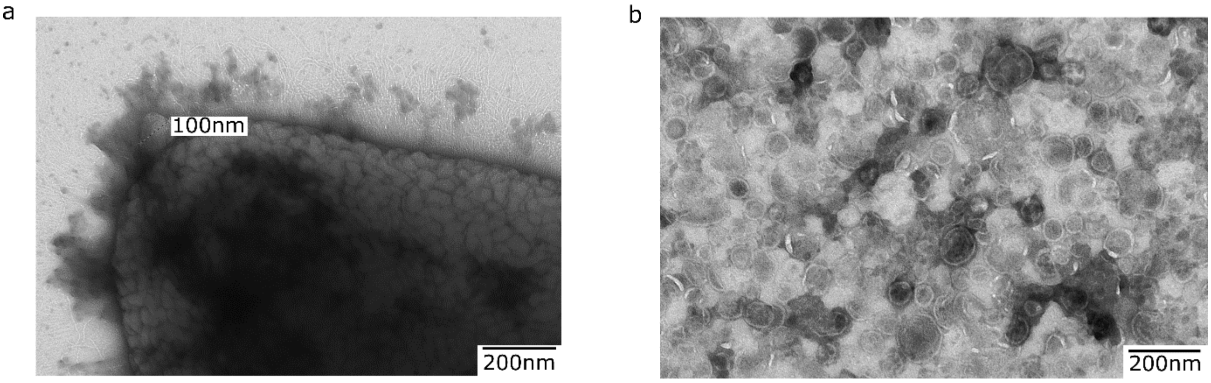

Supplementary Figure 8. TEM images of *C. indologenes #3362* (a) and isolated OMVs from *C. indologenes* culture supernatant (b). All image was taking using Tecnai TEM at 100 kV.

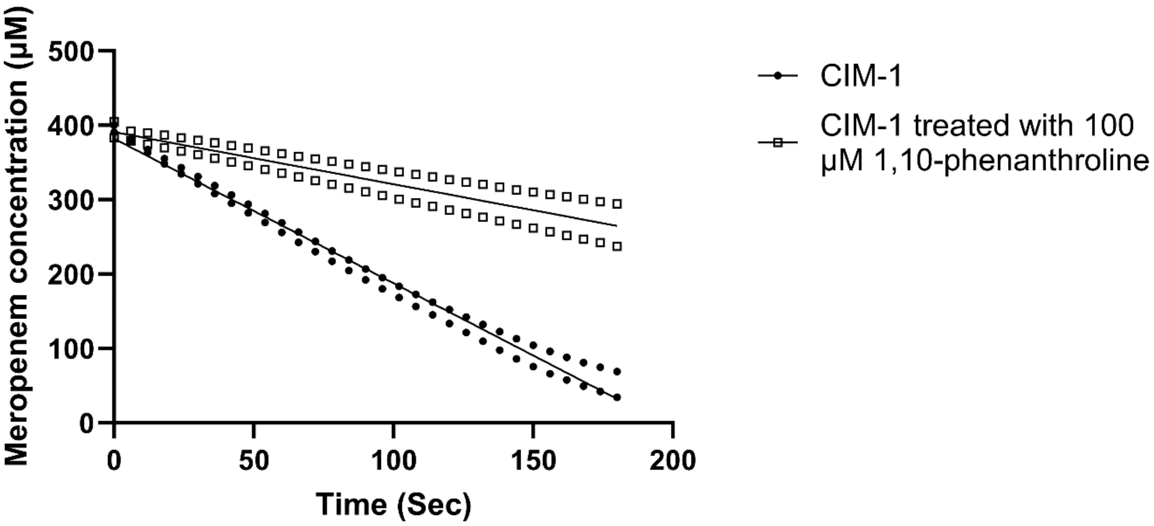

Supplementary Figure 9. The rate of hydrolysis of CIM-1ΔSig was significantly reduced at the present of zinc specific metal ion chelator, 1, 10-phenanthroline. 250 nm of CIM-1ΔSig was incubated with 100 µM of 1, 10-phenanthroline for 20 minutes. Absorbance at 320 nm was detected as an indication of unhydrolyzed meropenem substrates. Slope was calculated and compared between treated and untreated CIM-1ΔSig. n=2 independent experiments.

Two homologs from *Pedobacter* sp.

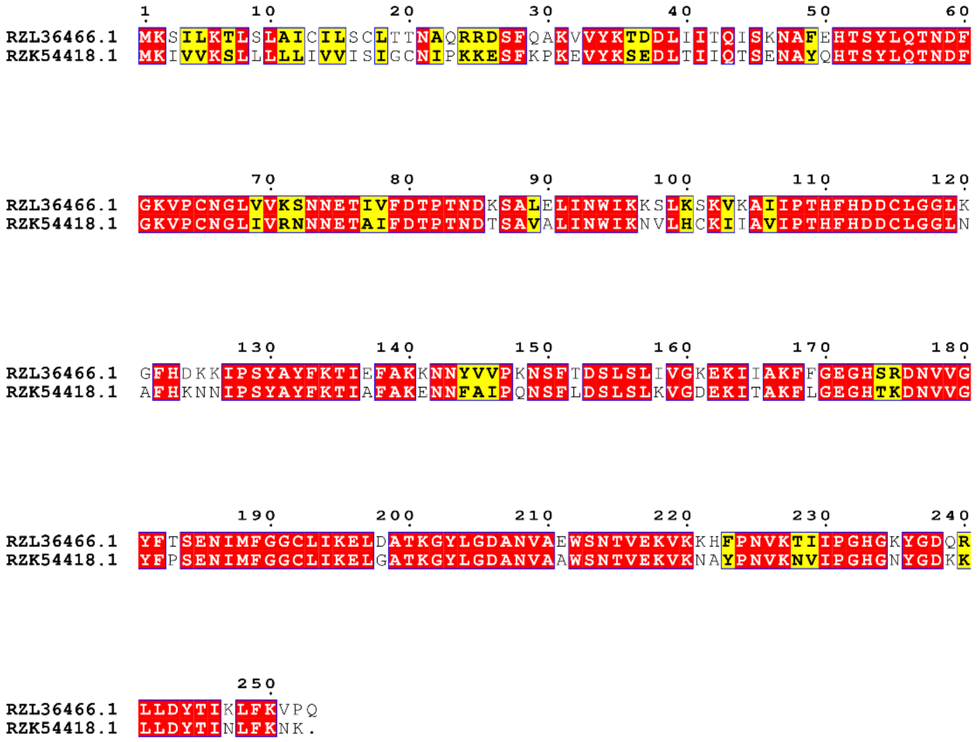

Two homologs from *Elizabethkingia anophelis*

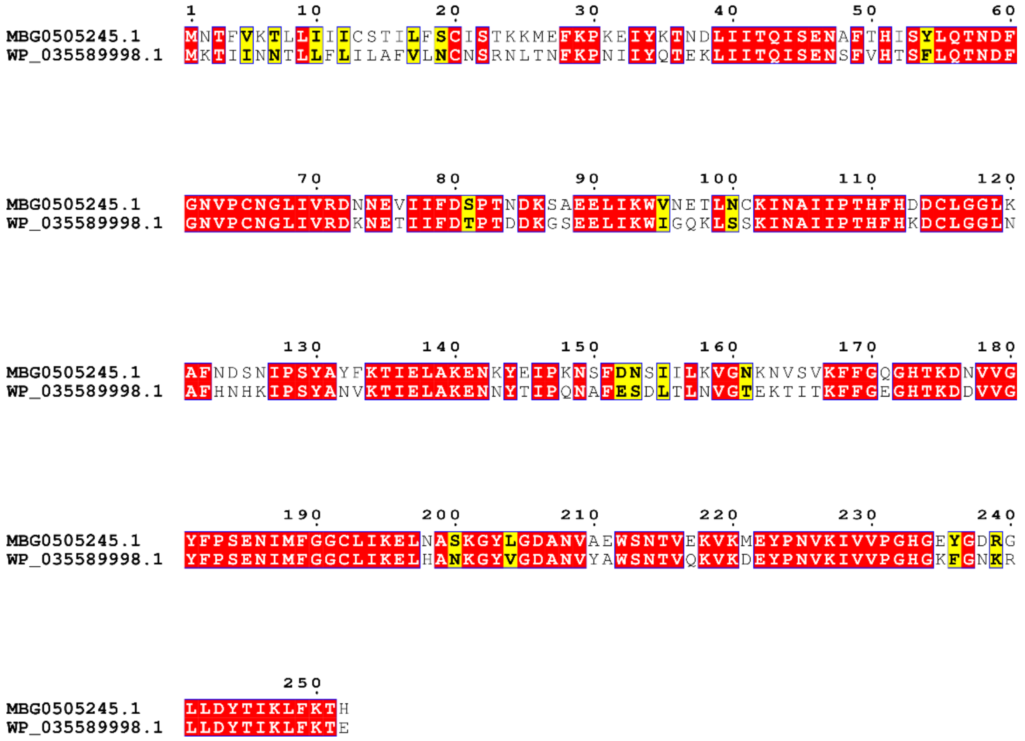

Supplementary Figure 10. Lower amino acid similarity in the signal peptides as comparison to the mature protein sequences was observed between the homologs, where ones are

predicted to be lipidated (MBG0505245.1 and RZK54418.1), the others are not. Identical amino acids are denoted as red box, similar amino acids across groups are denoted as yellow box.

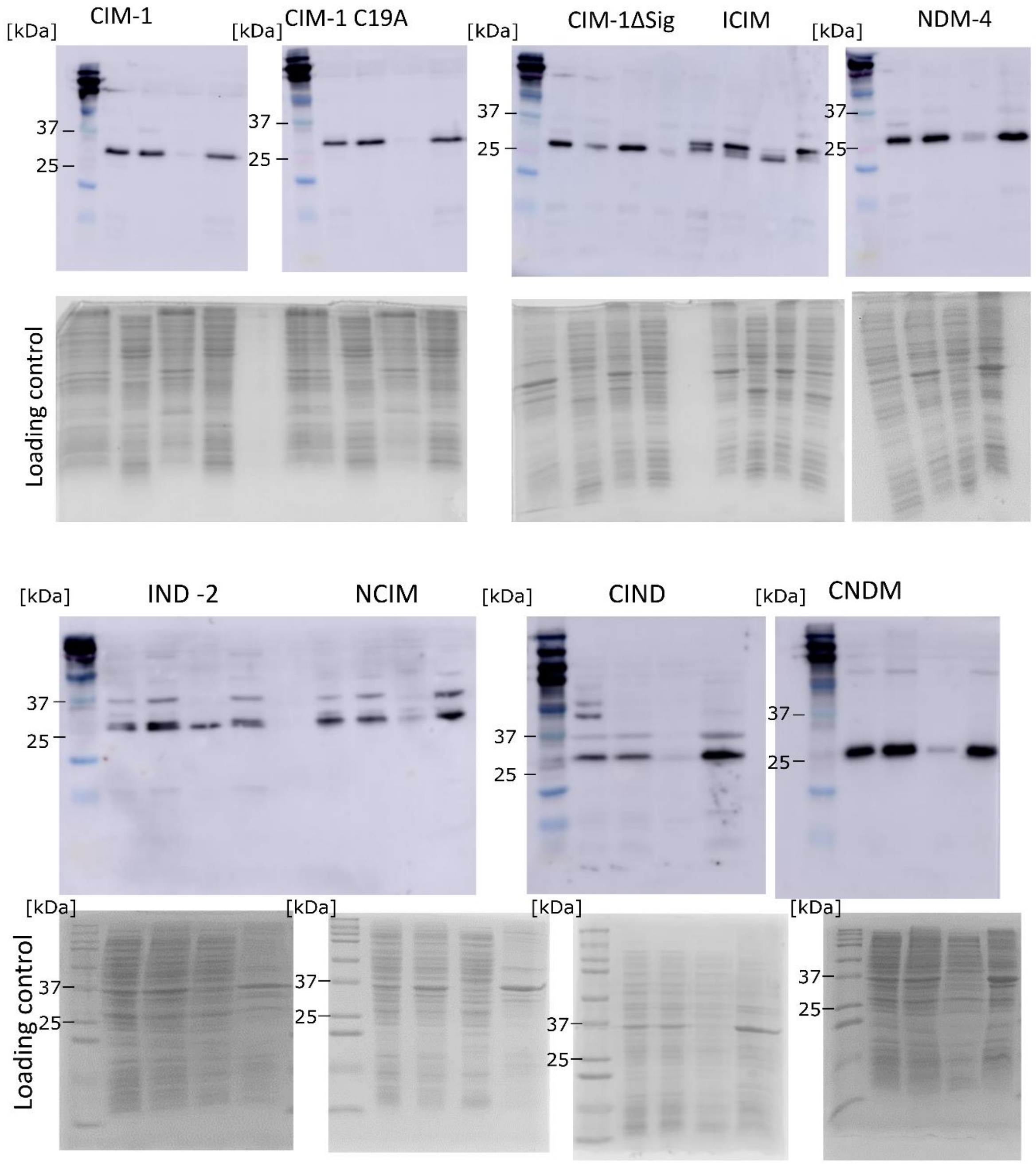

Supplementary Figure 11. Full, uncut gel images for Figure 3b. Precision Plus protein™ Kaleidoscope™ Pre-stained Protein marker (BIO-RAD) was used.

	gene	Ct1	Ct2	Ct3	Average Ct	DeltaCT	2^(-DeltaCt)	Expression level(-Fold)	Average expression level (-Fold)
**1**	***rho***	15.94	15.86	15.81	15.87				
	**bla_{CIM}**	21.1	21.06	21.02	21.06	5.19	0.03		
	**bla_{IND}**	17.21	17.33	17.47	17.34	1.47	0.36	13.21	
**2**	***rho***	16.4	16.27	16.27	16.31				
	**bla_{CIM}**	21.34	21.25	21.23	21.27	4.96	0.03		
	**bla_{IND}**	17.46	17.64	17.9	17.67	1.35	0.39	12.18	12.69

Supplementary Table 1. Relative expression level of CIM-1 and IND-2 in *C.* indologenes at the presence of 16 µg/mL meropenem. Experiment was repeated on two different days.
