# Supplementary material for: Identification and characterization of CIM-1, a carbapenemase that adds to the family of resistance factors against last resort antibiotics
Source: Commun Biol. 2024 Mar 7;7:282. doi: 10.1038/s42003-024-05940-0 (PMC10920655; doi:10.1038/s42003-024-05940-0)
Supplement: Supplementary file 3 — Description of Additional Supplementary Files [file 42003_2024_5940_MOESM3_ESM.pdf]

Description of Additional Supplementary Files

File name: Supplementary Data 1
Description: The source data behind the kinetic results (Table 3) in the paper.

File name: Supplementary Data 2
Description: The source data behind the mass spectrometry result (Table 2) in the paper.

File name: Supplementary Data 3
Description: The source data behind the phylogenetic tree (Figure 4) in the paper.
